# Elbow dislocation accompanied by ulnar coronoid process and radial head fractures: A report of a terrible triad injury in a child

**DOI:** 10.1016/j.radcr.2025.05.080

**Published:** 2025-06-26

**Authors:** Amir Bisadi, Fatemeh Abbasi, Mohammad Abbasalizadeh, Mahdi Mohammaditabar, Morteza Gholipour

**Affiliations:** a Bone Joint and Related Tissues Research Center, Akhtar Orthopedic Hospital, Shahid Beheshti University of Medical Sciences, Tehran, Iran; b Student Research Committee, Faculty of Medicine, Mazandaran University of Medical Sciences, Mazandaran, Iran; c Student Research Committee, Tabriz University of Medical Sciences, Tabriz, Iran; d Student Research Committee, School of Medicine, Alborz University of Medical Sciences, Karaj, Iran

**Keywords:** Pediatric, Elbow dislocation, Ulnar coronoid process, Radial head, Fracture, Terrible triad injury, Elbow injury

## Abstract

Elbow dislocation with fractures of the coronoid process and radial head, known as ``terrible triad injury'' (TTI), is a serious injury that is common in adults but rare in children. This report describes the case of a 9-year-old girl who experienced TTI after falling from a height of approximately 2 meters. She underwent closed reduction of the dislocation and had a K-wire inserted to stabilize her radial head, with removal after 3 weeks, followed by rehabilitation, resulting in satisfactory recovery of elbow joint motion at the 3-month follow-up. This case demonstrates that successful outcomes can be achieved through careful evaluation, appropriate imaging, and individualized treatment plans, while highlighting the need for prompt recognition and treatment to reduce complications like stiffness and arthritis in this rare pediatric injury pattern.

## Introduction

Elbow dislocation accompanied by fractures of the coronoid process and the radial head is commonly referred to as the ``terrible triad injury'' (TTI). This term highlights the challenges associated with managing this condition and the history of poor outcomes linked to it [1]. The TTI is a potentially severe and well-recognized injury in adults, frequently involving damage to the collateral ligaments. TTI typically leads to elbow instability, requiring surgical fixation if not treated appropriately [2,3]. The major goals of treatment are to restore the elbow's alignment for greater stability and facilitate early motion, which is critical for enhancing functional outcomes. Surgical stabilization and early mobilization are indicated for adults, and it's vital to highlight those desired clinical results can be attained when each component of the injury is successfully treated [4].

This condition is relatively uncommon in children, and there is ongoing debate regarding the effect of previous descriptions in the literature [[5], [6], [7]]. However, prompt recognition and appropriate intervention are crucial in minimizing complications such as stiffness, recurrent instability, and post-traumatic arthritis. The rarity of this injury in the pediatric population has resulted in a limited body of literature, making evidence-based decision-making more challenging. Yet, it emphasizes the significance of the surgical team's decision-making skills in such conditions.

We report the surgical management and functional outcomes of a 9-year-old girl who sustained a terrible triad injury following a fall. Fortunately, the patient maintained intact neurovascular function.

## Case presentation

The patient was a 9-year-old female who presented to our emergency department with a history of a 2-meter fall during play at a playground, resulting in trauma to the right elbow. The patient had no relevant past medical history or surgical history. She had all vaccinations up to date and no history of allergy. The patient had no history of a bleeding disorder, a connective tissue disorder, or a bone fragility syndrome.

On presentation, she had pain at the right elbow, 8 out of 10 on the pain scale, with extensive swelling and deformity. She was unable to move her elbow and had it held in a mildly flexed posture. Bruising and tenderness were noted over the lateral right elbow on examination. Neurovascular status was normal with a normal radial pulse, less than 2-second capillary refill, and an intact vascular supply and peripheral nerve function. Motor and sensory exam of the median, ulnar, and radial nerve distribution was intact. Range of movement testing was limited due to pain and instability.

**Fig. 1:**
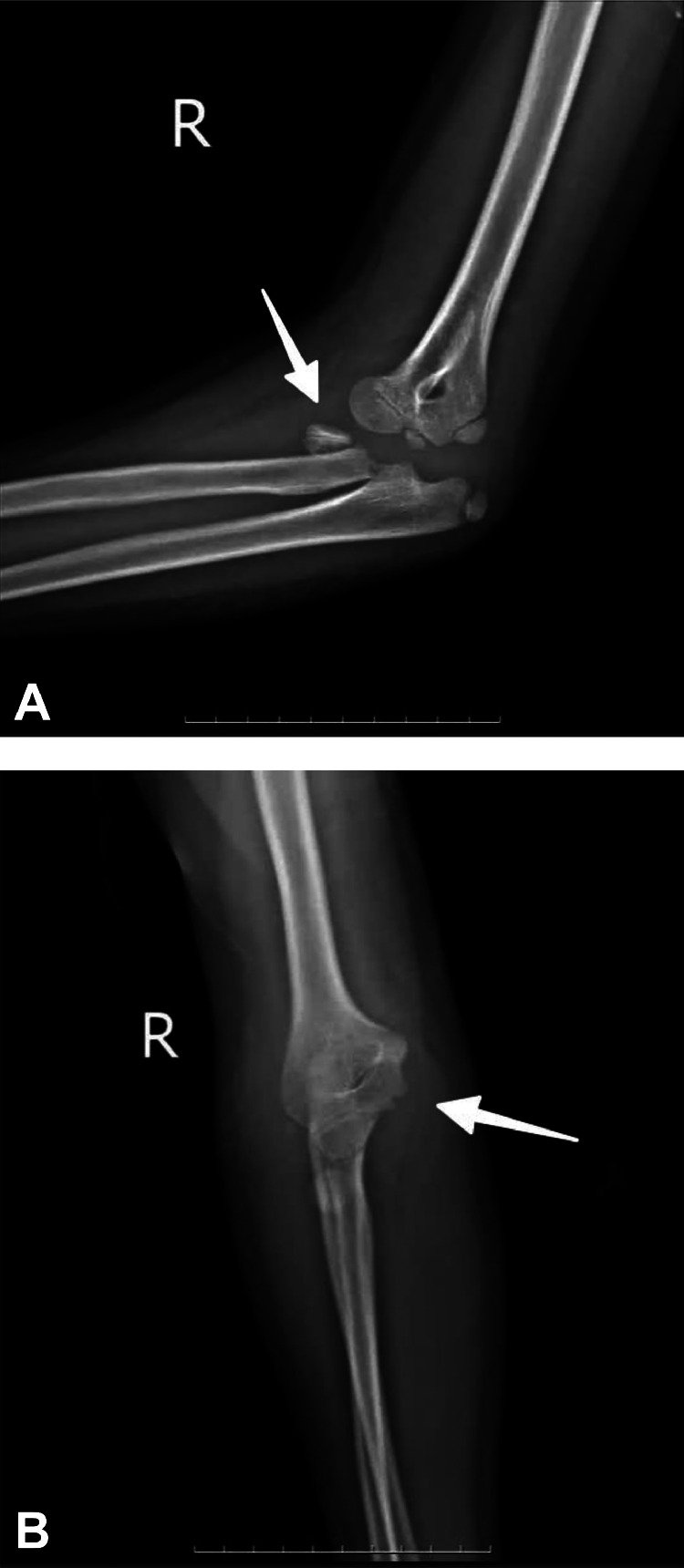
A: Initial radiographic evaluation of the right elbow Plain radiography (X-ray) in lateral view with the elbow flexed at approximately 90°, demonstrating posterior dislocation of the elbow joint with an associated radial head fracture (arrow). Note the loss of normal articulation between the distal humerus and the proximal radius and ulna, indicating posterior displacement of the forearm bones relative to the humerus. B: Initial radiographic evaluation of the right elbow Plain radiography (X-ray) in anteroposterior view with the elbow partially extended, showing medial displacement of the proximal radius and ulna. The radial head fracture is partially visualized from this view (arrow), with disruption of the normal radiocapitellar relationship.

Initial radiographic imaging (Fig. 1A and B) confirmed a displaced fracture involving the radial head.

**Fig. 2:**
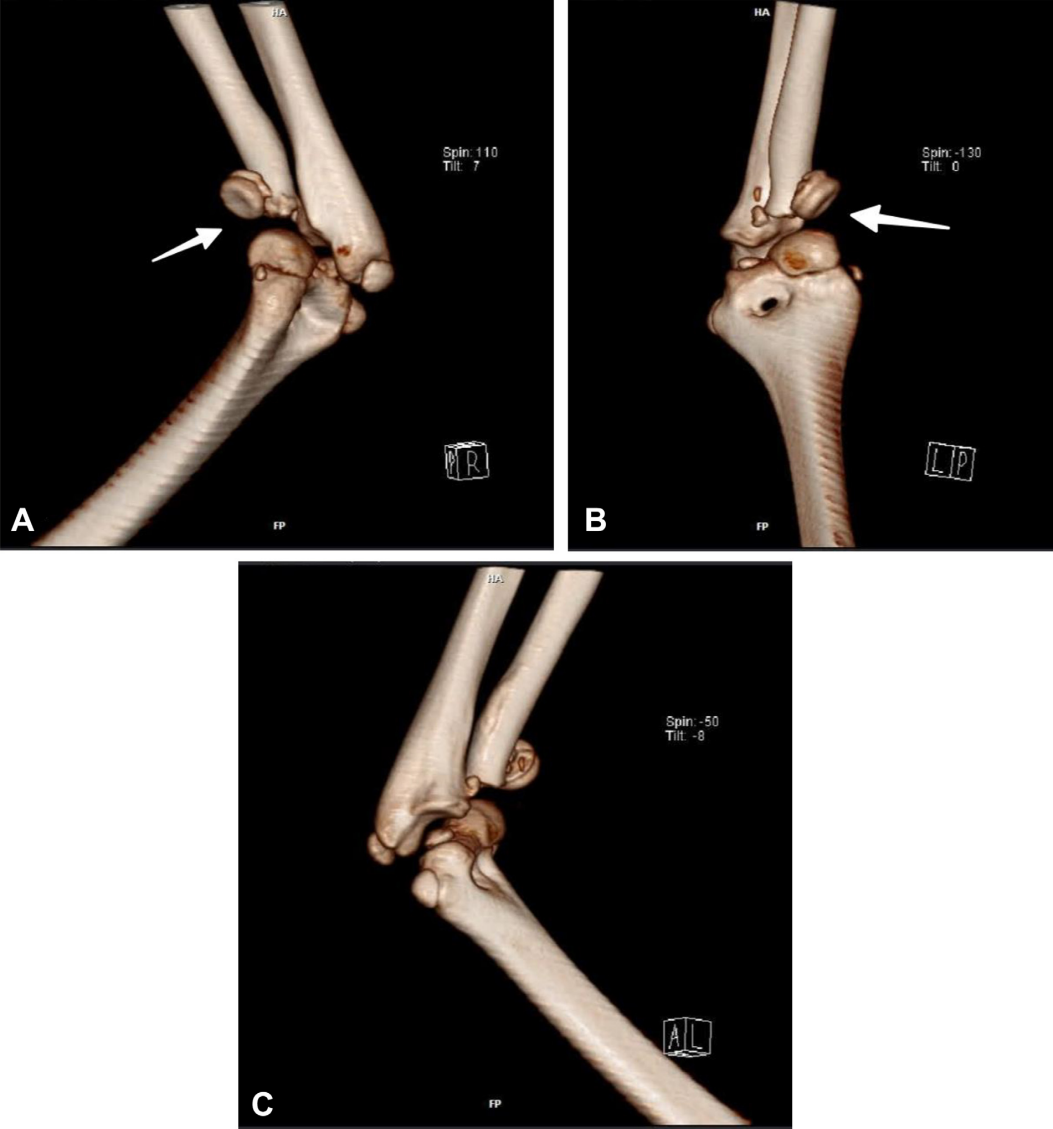
A: 3D CT reconstruction of the right elbow - lateral aspect Computed tomography with 3D volume rendering technique, lateral view, clearly demonstrating the three components of the terrible triad injury: (1) posterior dislocation of the elbow joint, (2) comminuted radial head fracture, and (3) coronoid process fracture. Note the posterior and lateral displacement of the radius and ulna relative to the distal humerus. B: 3D CT reconstruction of the right elbow - anterior aspect Computed tomography with 3D volume rendering technique, anterior view, providing enhanced visualization of the radial head comminution and coronoid process fracture (arrow). This perspective clearly demonstrates the anterior structural damage associated with the posterior dislocation mechanism. C: 3D CT reconstruction of the right elbow - medial aspect Computed tomography with 3D volume rendering technique, medial view, showing the relationship between the coronoid process fracture and the overall joint displacement. Note the integrity of the medial epicondyle despite the significant forces involved in this injury pattern.

**Fig. 3:**
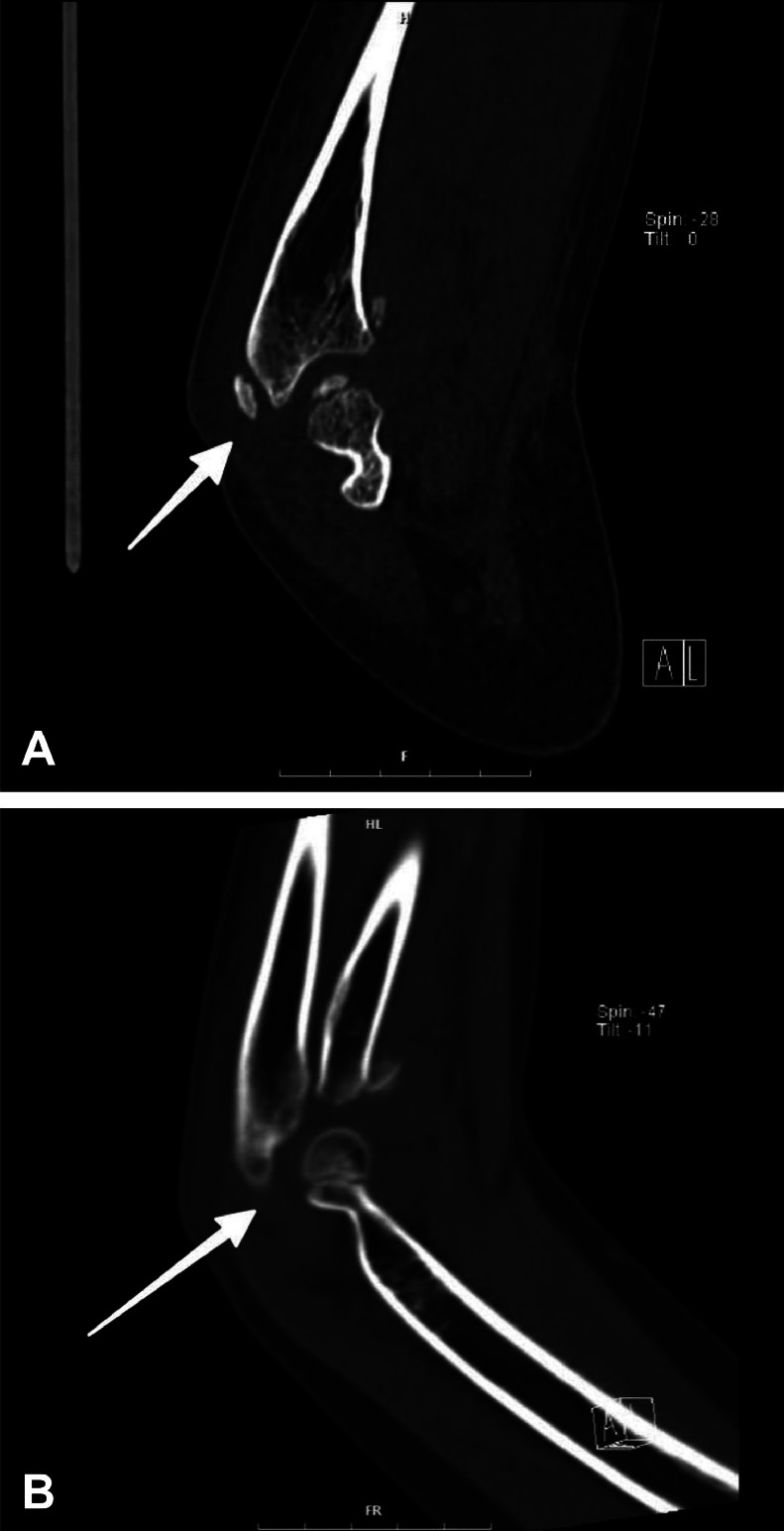
A: Axial CT image of the right elbow Computed tomography in axial plane, bone window, at the level of the radial head, demonstrating the comminuted radial head fracture (arrow) with multiple fragments. Slice thickness: 0.75mm, with 130 kVp settings. B: Sagittal CT image of the right elbow Computed tomography in the sagittal plane and bone window demonstrates the coronoid process fracture (arrow) and posterior subluxation of the radius and ulna. This image clearly shows the Type II coronoid fracture according to the Regan-Morrey classification.

3D and routine follow-up CT scans (Fig. 2A-C and Fig. 3A, B) gave great visualization of the terrible triad lesion, with representation of the coronoid process and radial head fracture, and posterior dislocation of the elbow joint. Laboratory results were within normal ranges with hematoglobin 12.4 g/dL (normal value range: 11.5-15.5 g/dL), white blood cell count 8.2 × 10³/µL (normal value range: 4.5-13.5 × 10³/µL), platelet count 265 × 10³/µL (normal value range: 150-450 × 10³/µL), and C-reactive protein 0.8 mg/L (normal value range: <1.0 mg/L).

**Fig. 4:**
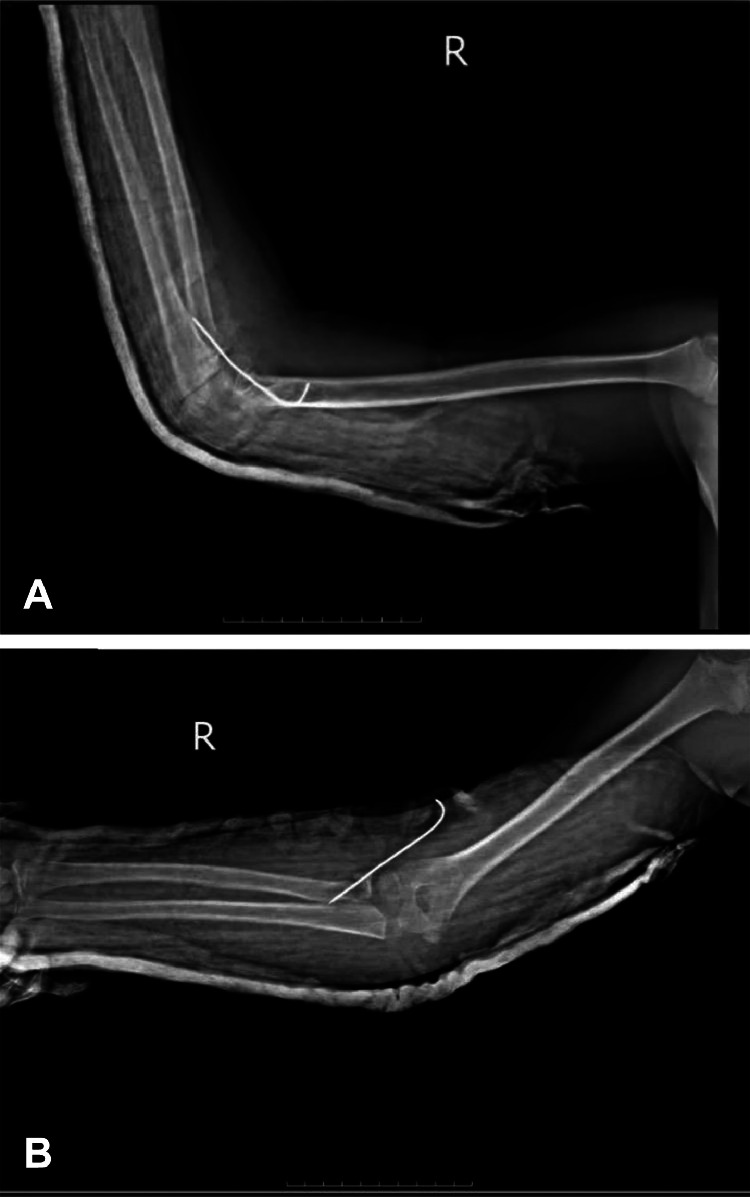
A: Post-operative lateral radiograph of the right elbow Plain radiography (X-ray) in lateral view with the elbow flexed at 90°, showing reduced elbow joint with K-wire fixation of the radial head (arrow). Note the restoration of normal articulation between the distal humerus and the proximal radius and ulna. B: Post-operative anteroposterior radiograph of the right elbow Plain radiography (X-ray) in anteroposterior view with the elbow partially extended, demonstrating appropriate K-wire placement through the radial head (arrow) and anatomic reduction of the joint.

Subsequently, there were extensive discussions with family members on options, risks, and benefits of treatments, and a final decision was made to proceed with surgical intervention. The patient underwent a regional axillary block for anesthesia and fluoroscopic-guided (C-arm) closed reduction. Reduction was accomplished by gentle longitudinal traction and sequential deformity correction. Internal stabilization was accomplished with a 1.6 mm K-wire for stabilization of the radial head. Postoperative radiographs (Fig. 4A and B) were satisfactory with excellent K-wire placement and acceptable reduction of the elbow joint. Intra-operative assessment on checking for restored stability indicated no instability throughout the arc of movement.

Postoperatively, the affected limb was immobilized with a cast at 90 degrees of flexion and neutral rotation. Postoperatively, she was put on appropriate analgesia and observation for any complication, most particularly compartment syndrome and neurovascular impairment. She was discharged on postoperative day 2 with instructions for rest, elevation, and follow-up.

**Fig. 5:**
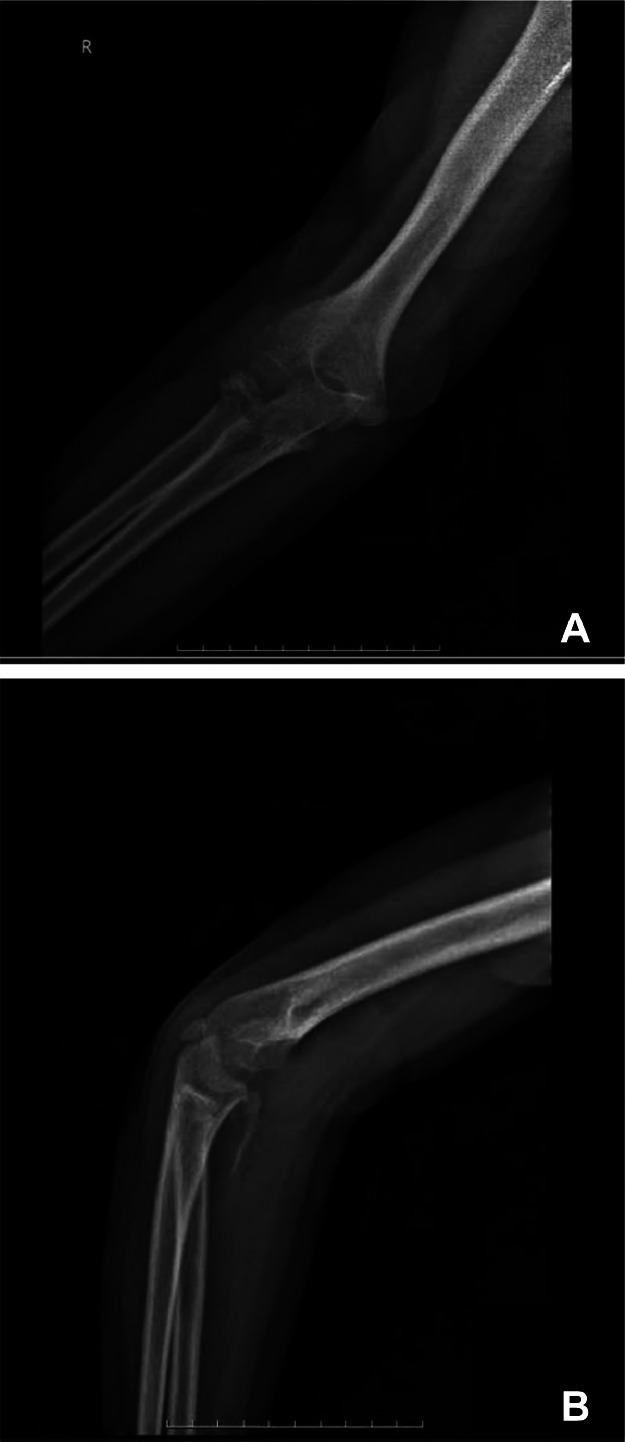
A: One-month follow-up anteroposterior radiograph of the right elbow Plain radiography (X-ray) in anteroposterior view, showing early healing of the radial head fracture following K-wire removal. Note the maintained joint reduction and improving fracture margins with early callus formation. B: One-month follow-up lateral radiograph of the right elbow Plain radiography (X-ray) in lateral view, demonstrating maintained reduction of the elbow joint after K-wire removal. The coronoid process and radial head show early signs of healing with no secondary displacement.

The patient had regular check-up visits to check the neurovascular status and heal the wound on a weekly basis. Follow-up radiographs a month back (Fig. 5A and B) indicated healing after K-wire removal. Three weeks later, on an outpatient basis, the K-wire was removed, and a rehabilitation regimen for regaining the range of motion (ROM) of the elbow joint started. The cast was fashioned into a removable splint to be worn during activities and removed for physiotherapy. The regimen of physiotherapy, with progressive passive followed by active exercises, with initial movements of flexion-extension supplemented later by exercises of pronation-supination, was tolerated by the patient. Physical visits were made thrice a week for a month and twice a week subsequently.

**Fig. 6:**
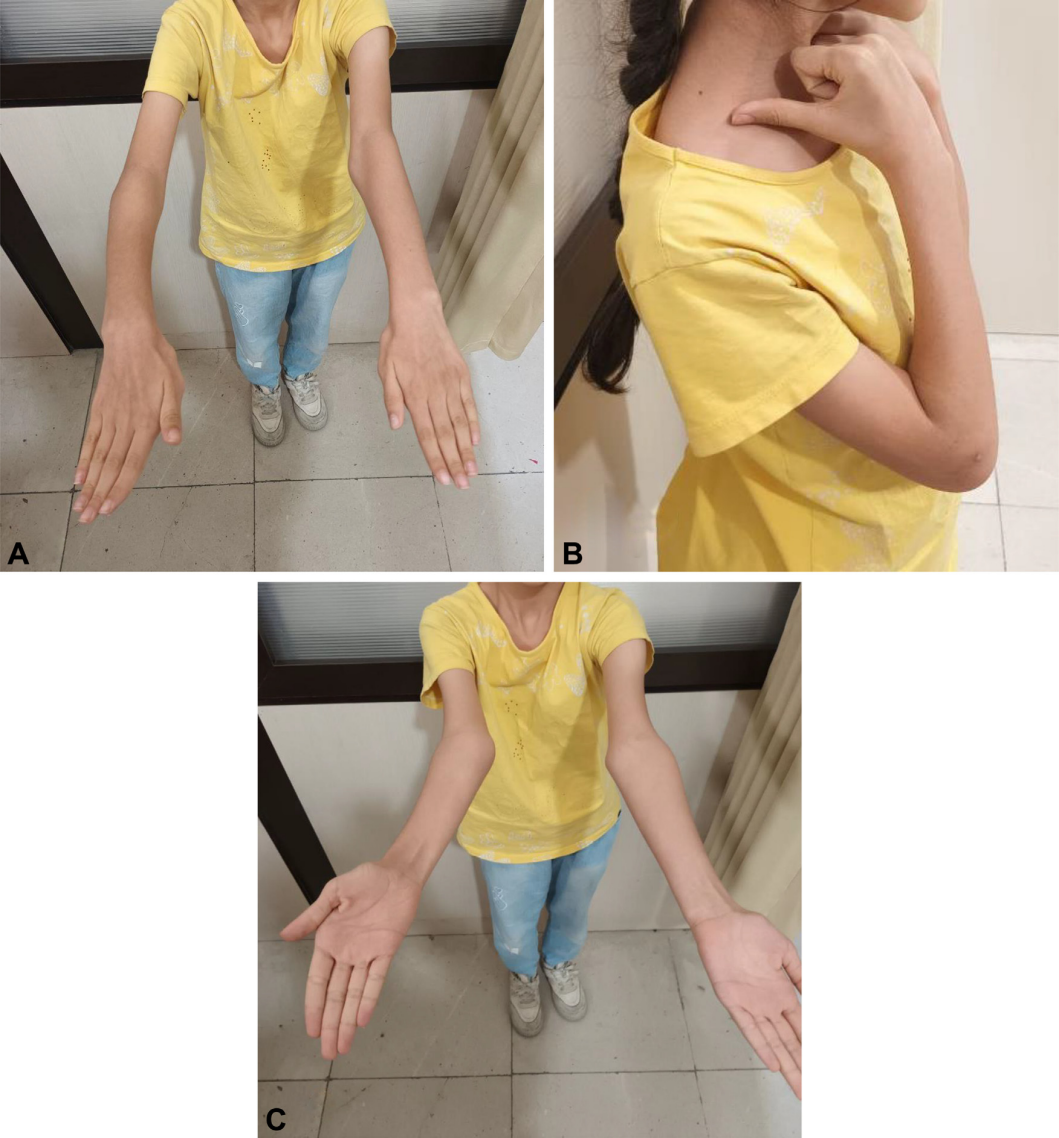
A: Clinical photographs at three-month follow-up Near-complete recovery of extension. B: Clinical photographs at three-month follow-up Flexion with a functional arc of approximately 135° (compared to 145° on the uninjured side). C: Clinical photographs at three-month follow-up Supination and pronation.

The patient returned for assessment at 3-month follow-up. Clinical and radiological assessment was carried out to assess healing, alignment, and function. The patient had excellent improvement, with satisfactory restoration of movement of the elbow joint, with 0-135 degrees of flexion (compared with 0-145 degrees on the normal side) and 70-0-80 degrees of pronation-supination (compared with 80-0-85 degrees on the normal side). Clinical photographs at 3-month follow-up (Fig. 6A-C) demonstrate restored range of movement and patient function. Mayo Elbow Performance Score was 90, which is excellent. Residual neurovascular compromise or any complication, eg, stiffness, nonunion, or malunion, did not occur. The patient had resumed nearly all activities of daily life, including school, though she was warned against playing any contact sport for an additional period of 3 months.

Follow-up care focused on ongoing rehabilitation to a maximal level of function and prevention of sequelae. The family and patient were instructed about adherence with exercise and complication detection. Follow-up within 6 months postinjury was scheduled for final assessment of full recovery and resolution of ongoing issues.

## Discussion

The so-called terrible triad injury (TTI) of the elbow is a complex pattern of injury including elbow dislocation with coronoid process or radial head fractures [5]. First described by Hotchkiss in 1996, the injury acquired a foreboding reputation based on a historically poor prognosis and difficult manageability [8]. Though TTI is well reported in adults, it is extremely rare in pediatrics, with scant cases reported within the literature [[5], [6], [7]]. Pediatric incidence of TTI is hard to define with high accuracy because of rarity, though injuries around the elbow are seen overall with 10%-12% of all fractures of childhood, with dislocations being far less common compared with isolated fractures [9].

The mechanics of TTI vary a bit among children due to ligament laxity and open growing plates. Supination of the forearm, valgus stress on the elbow, and a fall onto an outstretched, extended elbow hand cause the injury [5]. This mechanism produces relative posterior ulna displacement of the humerus with dislocation and fractures. In our patient, a female child of 9 years, who had fallen from a height of approximately 2 meters, consistent with what is reported in the literature, acquired the injury. Severe pain, swelling, and limitation of movement by the child are consistent with clinical presentation in reported cases of children [8].

Accurate diagnosis of TTI is attained through comprehensive imaging. Initial radiographs, while capable of showing obvious fractures and dislocations, underestimate the injury, especially with coronoid fractures [10,11]. In our case, initial radiographs revealed the dislocation and radial head fracture, with better delineation of involvement of the coronoid on subsequent CT scans. Advanced imaging studies are supported by literature for the diagnosis of TTI. 3D reconstructed CT scans are especially helpful for measurement of fragment size, displacement, and overall congruence of the joint, allowing for useful pretreatment assessment. MRI would provide additional information on ligamentous injury, although it has been avoided in our case due to obvious findings on CT and a lack of clinical presentation consistent with greater ligamentous compromise than with dislocation. Synthesis of clinical examinations with image findings is useful, allowing discrimination of non-accidental versus traumatic injury, which is confounding for the child [12,13].

Treatment of pediatric TTI is dynamic, with nonoperative or surgical being generally the principle of choice. Age, skeletal maturity, pattern of fractures, congruency of joints, and neurovascular status are all factors that must be taken into consideration when making a decision. In adults, operation would generally be preferred due to the inherent instability of injury. In children, due to wider healing potential and remodeling, a regimen of treatment could be varied [14]. Nonoperative treatment is an option for some cases with concentric reduction, minimal displacements, and congruency of joints. In a series reported by Baker et al., satisfactory values with nonoperative treatments were seen for carefully chosen individuals, with a mean Oxford Elbow Score of 46 and a range of movements of mean 131° flexion [15].

The primary aims of surgical TTI for kids are stabilization of the joints, repair/rebuilding of damaged tissues, and mobilization at an early stage [16]. In our case, successful operation with percutaneous K-wire stabilization and closed reduction of the radial head was sufficient for stabilization and reduction. Its minimally invasive process was appropriate for the age of the patient and the fractures. More invasive methods, such as open reduction and internal fixation (ORIF) or radial head arthroplasty (RHA), are, however, more commonly indicated for adult TTI. In a meta-analysis, Li et al. indicated RHA would attain more forearm rotational range of movement and fewer complications compared with ORIF for adult cases [14], although replacing the radial head is normally avoided in kids due to growing complications.

Rehabilitation after TTI treatment is crucial for optimizing function. The timing of mobilization is a trade-off between the risk of stiffness with immobilization for an extended period of time and the risk of instability with too prompt movement [17]. Postimmobilization for 3 weeks after K-wire removal, our patient started rehabilitation with an increasing regimen with a focus on restitution of movement. This is supported by recommendations by Corbet et al., since there is an excellent outcome (mean Mayo Elbow Performance Score 89.1) with early mobilization with effective surgical stabilization [16]. Our patient had an excellent outcome with a Mayo Elbow Performance Score of 90 over a period of 3 months, with almost complete restitution of function and movement.

Complications of TTI include recurrent instability, stiffness, heterotopic ossification, post-traumatic arthritis, and growth disturbance [17]. Fortunately, all of these complications were avoided in our patient, a tribute to prompt diagnosis, proper management, and strict adherence to rehabilitation protocol. The favorable outcome of the present case attests to the importance of tailored regimens based on unique characteristics of injury and patient-specific parameters, as supported by Waterworth et al., based on a description of present concepts of TTI management [17].

## Conclusion

The management of TTI in children requires a multifaceted approach, including meticulous preoperative evaluation, the use of advanced imaging modalities, and individualized treatment plans. The presented case demonstrates the successful application of these principles, resulting in a favorable outcome for the patient. Further research and case reports are needed to enhance our understanding and management of this complex injury pattern in the pediatric population.

## Patient consent

Written informed consent was obtained from the patient for publication and any accompanying images. A copy of the written consent is available for review by the Editor-in-Chief of this journal on request.
